# The metastability of γ‐secretases as foundation for AD onset and prevention

**DOI:** 10.1002/alz70855_098879

**Published:** 2025-12-23

**Authors:** Lucia Chávez‐Gutiérrez

**Affiliations:** ^1^ VIB‐KULeuven, Leuven, Vlaams Brabant, Belgium

## Abstract

**Background:**

Autosomal Dominant Alzheimer's Disease (ADAD), caused by mutations in the PSEN1/2 and APP genes, shows variable age at symptom onset (AAO) across mutation types, providing a model for understanding disease mechanisms.

**Method:**

We assessed γ‐secretase dysfunction by comprehensive Aβ profiling of 28 PSEN2 and 18 APP mutations, using PSEN1/2‐deficient or HEK293 cells. We analyzed Aβ profile‐AAO relationships, predicted (biochemical) AAOs, and compared clinical and biochemical data. In addition, we investigated the stability of enzyme‐substrate (E‐S) complexes using cell‐free and cellular assays.

**Results:**

Mutation‐induced shifts in Aβ ratios correlated linearly with AAO. Integration with PSEN1 data revealed parallel but shifted linear Aβ‐AAO correlations. In addition, our analyses show that 'signature' Aβ profiles are largely explained by differential E‐S stability.

**Conclusion:**

Our study supports a unified model of AD pathogenesis, in which mutation‐mediated destabilization of γ‐secretase leads to its dysfunction and shifts Aβ profiles towards generation of longer peptides. The molecular composition of (mutant) Aβ profiles largely determines AAO in carriers of mutations in the causal genes. This emphasizes the pathogenicity of Aβ profile imbalances, rather than specific peptide elevations. Our findings offer potential for predictive AAO modeling with implications for clinical and genetic research, and support γ‐secretase targeting strategies in ADAD therapy and potentially more broadly in AD.

